# Integrated analysis reveals the pivotal interactions between immune cells in the melanoma tumor microenvironment

**DOI:** 10.1038/s41598-022-14319-2

**Published:** 2022-06-16

**Authors:** Jiawei Chen, Shan Hu, Huiqi Wang, Tingxiu Zhao, Yue Song, Xueying Zhong, Qingling Luo, Mansi Xu, Lina He, Qiugu Chen, Biaoyan Du, Jianyong Xiao, Kun Wang

**Affiliations:** 1 Research Center of Integrative Medicine, School of Basic Medical Sciences, Guangzhou University of Chinese Medicine, Guangzhou, 510006 China; 2 Department of Pathology, Guangzhou University of Chinese Medicine, Guangzhou, 510006 China; 3 Department of Biochemistry, Guangzhou University of Chinese Medicine, Guangzhou, 510006 China

**Keywords:** Cancer, Computational biology and bioinformatics, Genetics, Immunology

## Abstract

Melanoma is the most lethal type of skin cancer. Despite the breakthroughs in the clinical treatment of melanoma using tumor immunotherapy, many patients do not benefit from these immunotherapies because of multiple immunosuppressive mechanisms. Therefore, there is an urgent need to determine the mechanisms of tumor-immune system interactions and their molecular determinants to improve cancer immunotherapy. In this study, combined analysis of microarray data and single-cell RNA sequencing data revealed the key interactions between immune cells in the melanoma microenvironment. First, differentially expressed genes (DEGs) between normal and malignant tissues were obtained using GEO2R. The DEGs were then subjected to downstream analyses, including enrichment analysis and protein–protein interaction analysis, indicating that these genes were associated with the immune response of melanoma. Then, the GEPIA and TIMER databases were used to verify the differential expression and prognostic significance of hub genes, and the relationship between the hub genes and immune infiltration. In addition, we combined single cell analysis from GSE123139 to identify immune cell types, and validated the expression of the hub genes in these immune cells. Finally, cell-to-cell communication analysis of the proteins encoded by the hub genes and their interactions was performed using CellChat. We found that the CCL5-CCR1, SELPLG-SELL, CXCL10-CXCR3, and CXCL9-CXCR3 pathways might play important roles in the communication between the immune cells in tumor microenvironment. This discovery may reveal the communication basis of immune cells in the tumor microenvironment and provide a new idea for melanoma immunotherapy.

## Introduction

Melanoma (skin cutaneous melanoma (SKCM)) is the most aggressive and poorly prognosed skin cancer, accounting for more than 80% of skin cancer-related deaths. The incidence of SKCM has continued to rise in recent years, posing a serious threat to human life and health^1,2^. The immune system has been shown to have anti-tumor capabilities in a variety of malignancies (e.g., melanoma and lung cancer)^3^. Previous studies have identified multiple types of tumor infiltrating lymphocytes (TILs) in the tumor microenvironment (TME), which provide all the metabolites and factors that control tumor cell proliferation, dissemination, and drug resistance^4^. Melanoma has traditionally been considered an immunogenic malignancy^4^, and the TME significantly influences the diagnosis, survival outcome, and clinical management of patients with melanoma. The distribution and density of TILs not only affect the survival of patients with melanoma^5^, but also regulate the progression of melanoma^3^. In this study, we aimed to explore more sensitive and efficient biomarkers to improve the treatment and prognosis of melanoma. Based on these biomarkers, we also aimed to reveal the potential molecular mechanism of immune cell interaction in the TME to improve immunotherapy for patients with melanoma.

With the rapid development of high-throughput technologies, bulk RNA sequencing (RNA-seq) technologies have been used widely in gene expression research at the population level. In recent years, single-cell sequencing technologies have also provided the possibility to explore gene expression profiles at the single-cell level^6^. Single-cell sequencing allows high-throughput sequencing analysis of the genome, transcriptome, and epigenome of individual cells, reflecting intercellular heterogeneity, and gene and molecular functional diversity. However, to explain the mechanisms of disease occurrence at the molecular level requires a deeper exploration of the data. Jin et al. developed CellChat, a tool capable of quantitatively inferring and analyzing intercellular communication networks from single cell RNA sequencing data^7^. Cellular communication is the process by which cells receive, handle, and transmit signals from other surrounding cells or themselves, which plays an important role in coordinating various biological processes.

In this study, a protein–protein interaction (PPI) network was constructed based on differentially expressed genes (DEGs) from The Gene Expression Omnibus GEO database sets GSE15605 and GSE114445. Finally, IDO1(Indoleamine 2, 3-dioxygenase 1), SELL(L-selectin), FCGR2A(Fc gamma receptor IIa), GZMB(granzyme B), CD27(CD27 molecule), CXCL9(C-X-C motif chemokine ligand 9), ICAM1(intercellular adhesion molecule 1), CCL4(C–C motif chemokine ligand 4), CCL5(C–C motif chemokine ligand 5), CXCL10(C-X-C motif chemokine ligand 10), and CTLA4 (cytotoxic T-lymphocyte associated protein 4) were identified as significant predictive biomarkers for melanoma. We also combined single cell sequencing technology with CellChat to predict the intercellular communication and analyzed the potential interaction between immune cells in the TMR. We found that CCL5-CCR1, SELPLG-SELL, CXCL10-CXCR3, and CXCL9-CXCR3 interactions might form the basis of two-way communication between immune cells in the TME.

## Results

### Identification of DEGs

**Figure 1:**
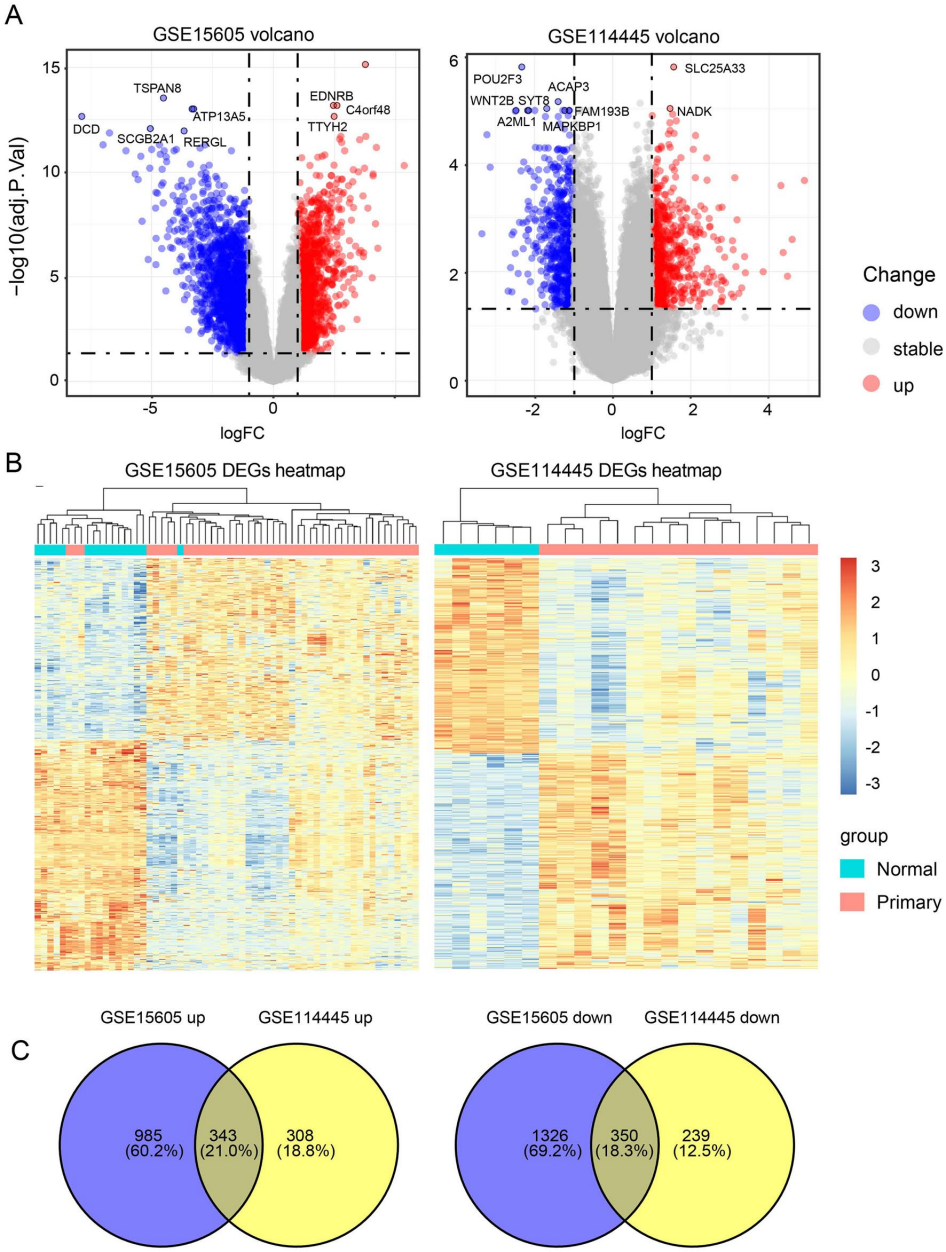
Screening results of DEGs. (**A**) A volcano map of DEGs in the GSE15605 and GSE114445 data sets. The red, blue, and gray points represent genes that were upregulated, downregulated, and showed no significant differences in expression, respectively. (**B**) Heat map of differential gene expression in the GSE15605 and GSE114445 datasets. (**C**) A Venn diagram displaying the number of DEGs in the two datasets from the GEO database. *DEG* differentially expressed gene, *down* downregulated, *up* upregulated, *stable* no difference in expression.

GEO2R analysis identified 3004 DEGs in GSE15605 and 1240 DEGs in GSE114445 (Supplementary file 1). To visualize the distribution of the DEGs, we obtained expression matrices from GEO to plot volcano and heat maps using the ggplot2 package (Fig. 1A,B). The overlap of two datasets contained 343 upregulated genes and 350 downregulated genes, as shown in the Venn plot (Fig. 1C).

### GO enrichment analysis and KEGG pathway analysis of the DEGs showed functional enrichment in immune regulation

**Figure 2:**
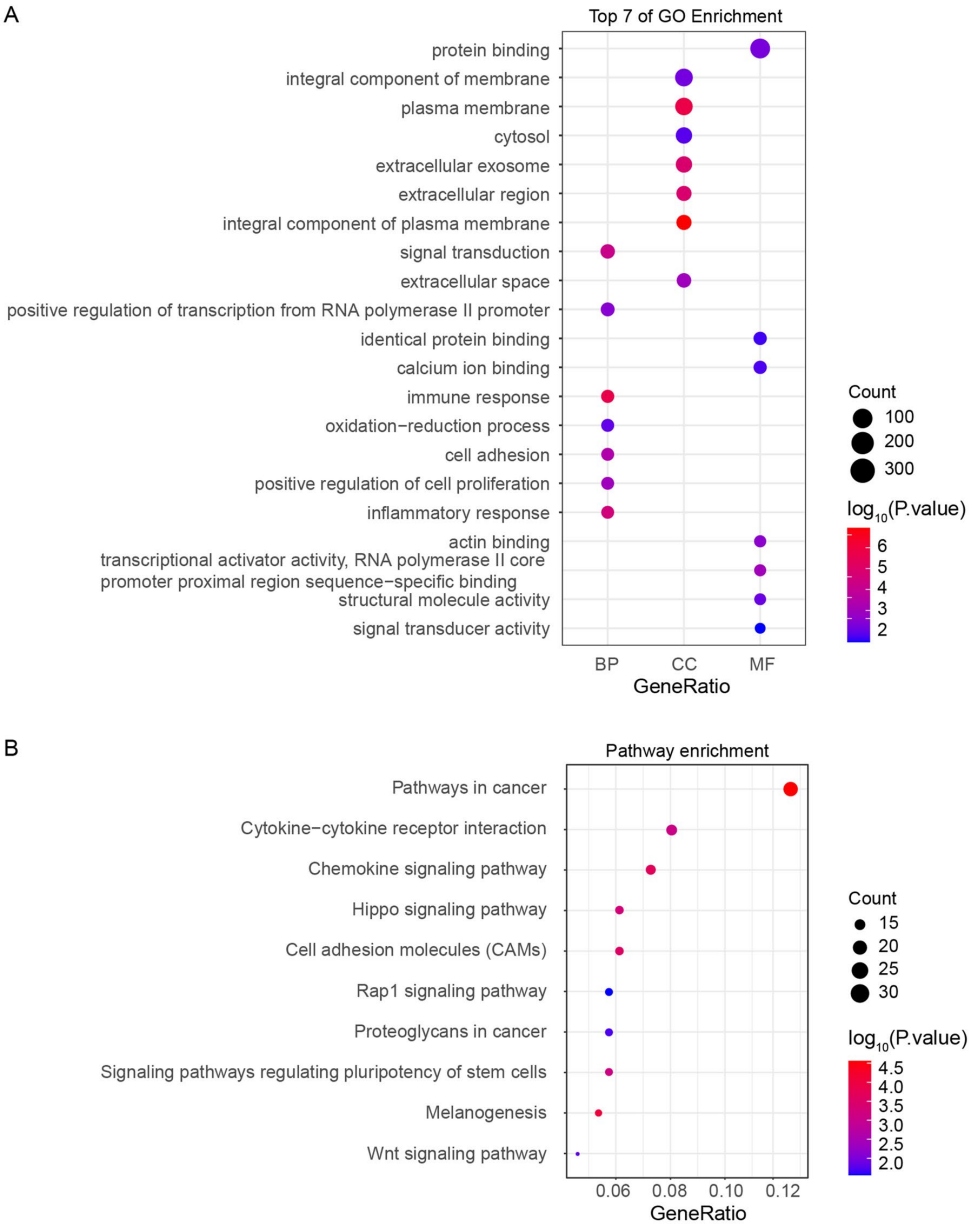
GO and KEGG pathway enrichment analysis of the DEGs. (**A**) GO analysis of DEGs, including biological processes, cell components, and molecular functions. (**B**) KEGG pathway enrichment analysis of the DEGs. The GO analysis and KEGG pathway enrichment analysis of the DEGs were completed using DAVID and visualized using the ggplot2 package. *GO* gene ontology, *KEGG* Kyoto Encyclopedia of Genes and Genomes, *DEG* differentially expressed gene.

To explore the potential signaling pathways and biological functions involving the common DEGs, we used the DAVID database to perform GO annotation and KEGG pathway enrichment analyses, which were visualized using ggplot2 to produce bubble plots (P < 0.05, Fig. 2). GO analysis identified that the DEGs were mainly associated with biological processes such as immune response, inflammatory response, and signal transduction. In molecular function, the main changes focused on protein binding and transcription activators. The changes in cellular component were mostly plasma membrane, plasma membrane components, and extracellular secretion (Fig. 2A). Figure 2B shows that the top five KEGG-enriched pathways associated with the DEGs were cancer pathway, cytokine receptor interaction, chemokine signaling pathway, Hippo signaling pathway, and cell adhesion molecules (CAMs), and are enriched in immune responses, cytokine receptor interactions, and chemokine-mediated signaling pathways. These results showed that DEGs play an important role in cancer development of patients, which is related to the activation status of immune cells in the TME and the state of the immune response induced by various cytokines in the body, suggesting that these DEGs might be the key factors in the regulation of immune processes in the TME.

### PPI network construction and the identification of hub genes

**Figure 3:**
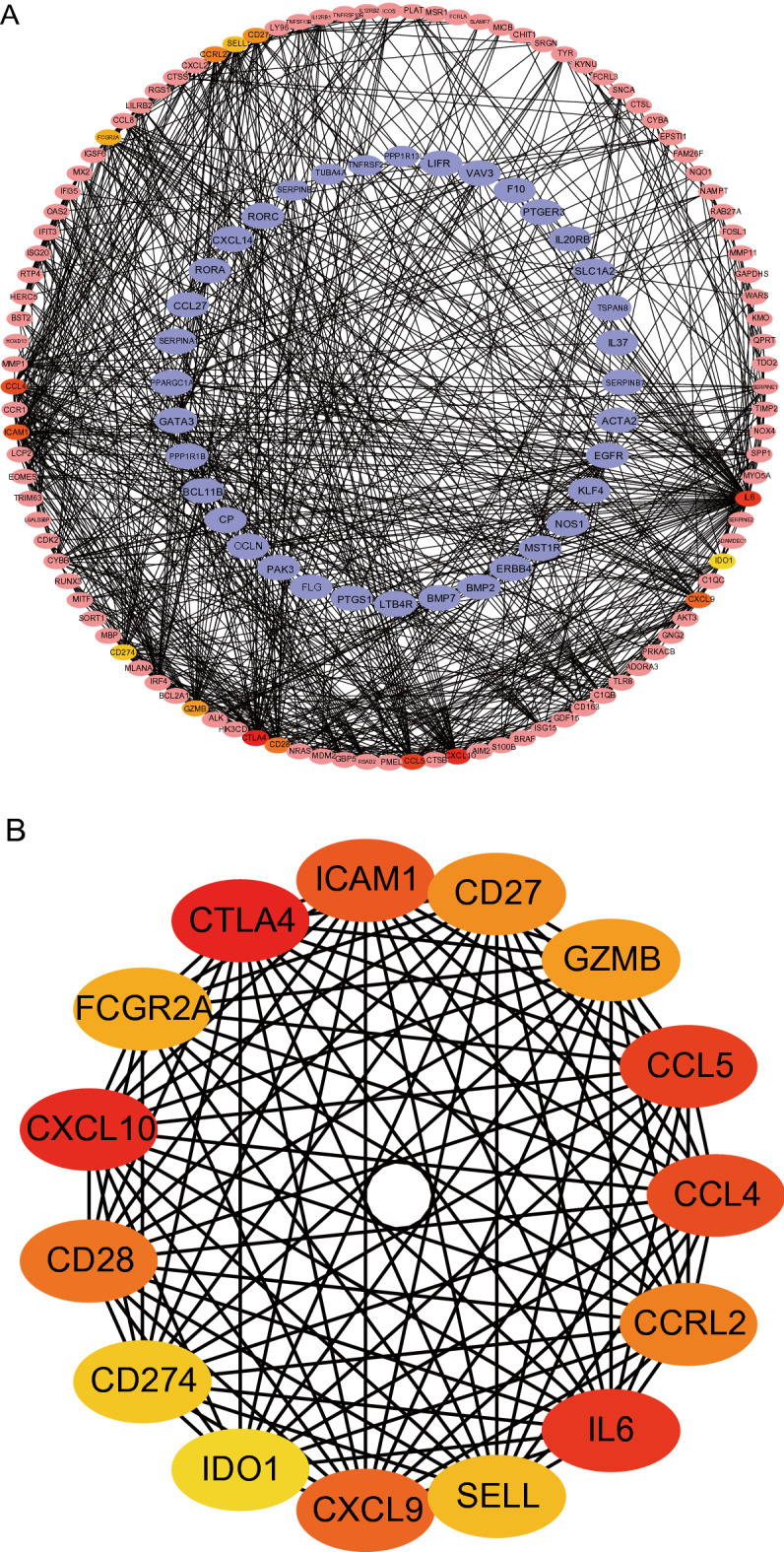
Construction of a PPI network and the identification of hub genes. (**A**) PPI network of DEGs. The outer circle represents upregulated genes, The outer circle represents downregulated genes, and the darker the color, the stronger the connection between the gene and other genes. (**B**) Top 15 DEGs obtained by MCC of cytohubba. *DEG* differentially expressed gene, *PPI* protein–protein interaction, *MCC* maximal clique centrality.

To further investigate the interaction of the identified DEGs, we constructed a Protein–protein interactions (PPI) network using STRING and Cytoscape to explore the interactions and central genes of DEGs. A total of 693 DEGs were uploaded to the STRING website and the comprehensive score > 0.4 was used as the cut-off standard (Supplementary file 2). First, the PPI network was constructed using Cytoscape (Fig. 3A), which included 618 nodes and 2289 edges. Nodes represent proteins, edges represent interactions between proteins, and the number of edges connected by genes is positively correlated with the importance of their functions in the PPI network. Additionally, we showed the PPI networks of genes with a score threshold of 10,000 in Fig.S1 to observe genes with higher connectivity. And then the top 15 hub genes with high connectivity in the PPI network were determined using the plug-in maximal clique centrality (MCC) of cytohubba: CCRL2, CD28, CD274, IL6, CTLA4, CXCL10, CCL5, CCl4, ICAM1, CXCL9, CD27, GZMB, FCGR2A, SELL, and IDO1 (Fig. 3B). Top 15 hub genes are all upregulated DEGs, and their higher scores suggest that they may play an important role in the development of melanoma and could be a key target in the treatment of melanoma (Supplementary file 3).

### Expression verification and survival analysis of the hub genes

**Figure 4:**
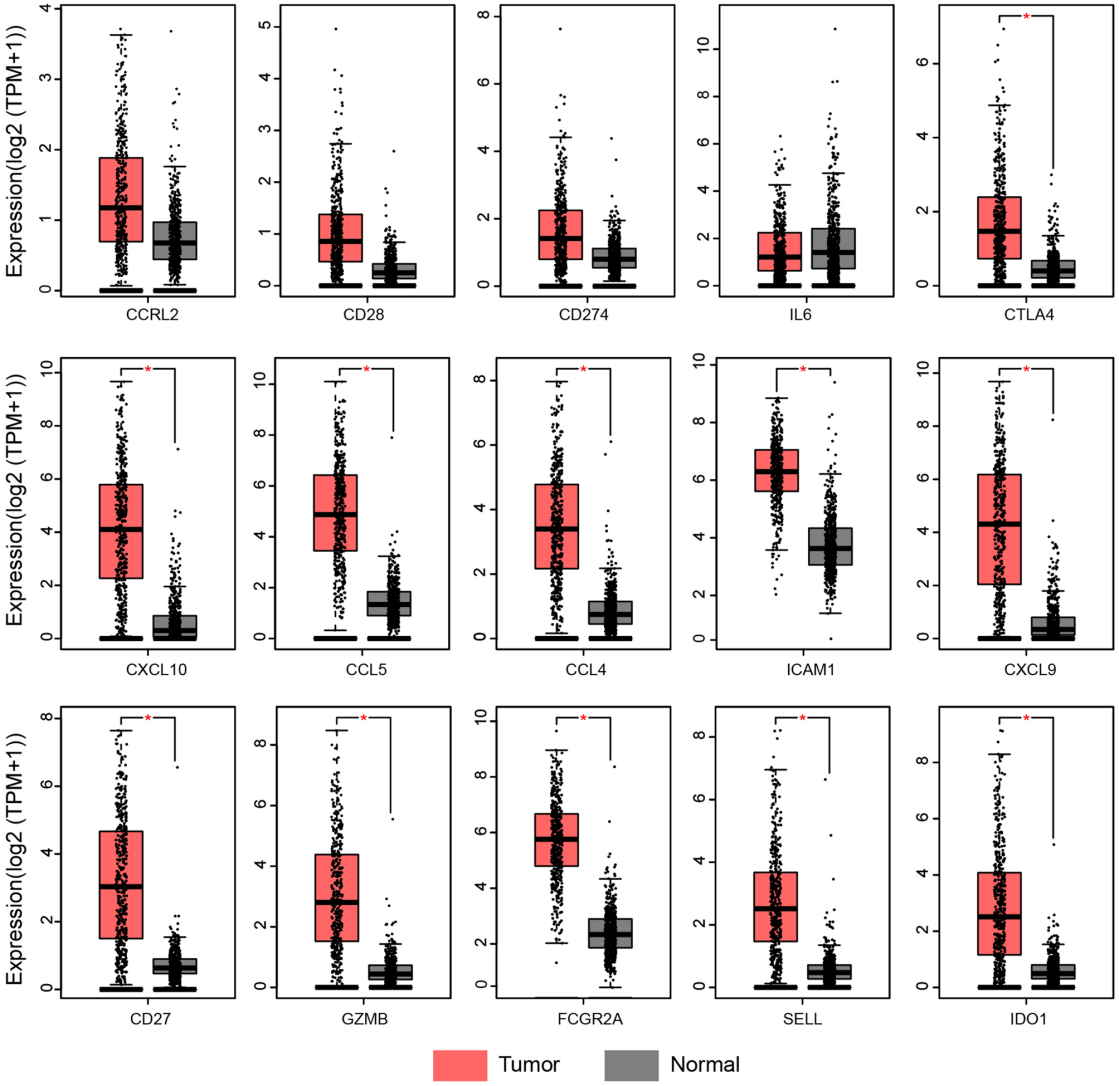
Expression level of hub genes in SKCM compared with normal tissues. Melanoma was compared with normal tissues in the GEPIA database, red represents tumor tissue and gray represents normal tissue. *SKCM* skin cutaneous melanoma.

To investigate the expression of hub genes screened from the above datasets in other SKCM and normal tissue samples, we analyzed the RNA-seq data of SKCM and normal skin tissues from the TCGA and GETx databases through the GEPIA database, including a total of 461 tumor samples and 558 normal samples. The results showed that CCRL2, CD28, CD274, and IL6 were up-regulated in SKCM, but the difference was not statistically significant (P > 0.05). The expression levels of other hub genes CTLA4, CXCL10, CCL5, CCl4, ICAM1, CXCL9, CD27, GZMB, FCGR2A, SELL, IDO1 in SKCM were higher than those in non-tumor skin tissues (P < 0.05, Fig. 4), and the results were statistically significant.

### Prognostic value of differentially expressed hub genes in patients with melanoma

**Figure 5:**
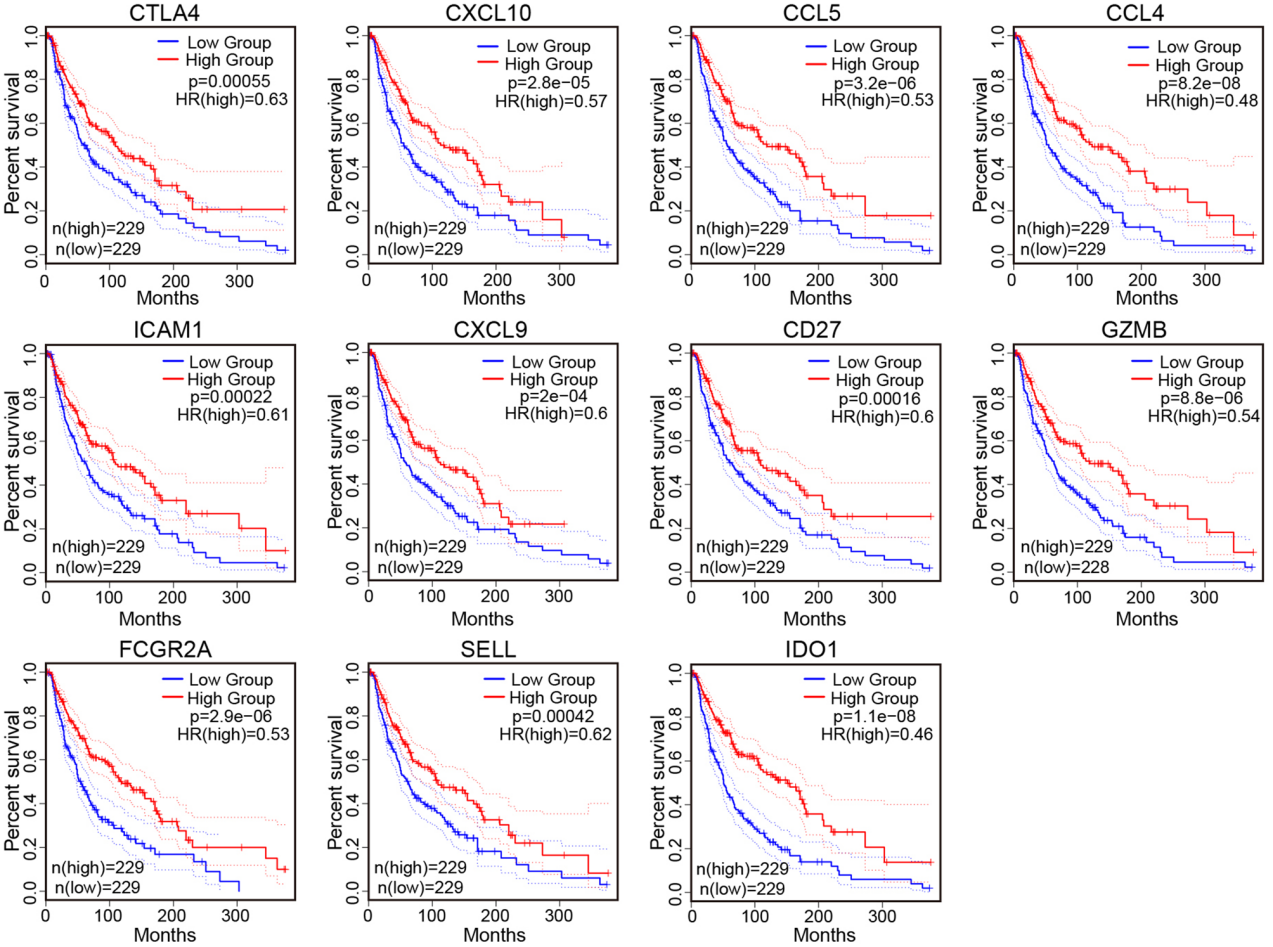
Prognostic value of hub genes. Kaplan–Meier survival curves comparing the relationship between high and low expression of hub genes in the GEPIA database and overall survival in SKCM patients, the red curve represents high expression levels, while the blue curve represents low expression levels. *SKCM* skin cutaneous melanoma.

To study the prognostic value of these differentially expressed hub genes in melanoma, we performed Kaplan–Meier survival analysis using GEPIA. According to the median value of hub genes expression, SKCM patients were divided into high expression group and low expression group. The results showed that the high expression levels of 11 hub genes correlated significantly with longer overall survival (Fig. 5): CTLA4 (logrank p = 0.00055, hazard ratio (HR) = 0.63), CXCL10 (logrank p = 2.8e−05, HR = 0.57), CCL5 (logrank p = 3.2e−06, HR = 0.53), CCL4 (logrank p = 8.2e−08, HR = 0.48), ICAM1 (Logrank p = 0.00022, HR = 0.61), CXCL9 (logrank p = 2e−04, HR = 0.6), CD27 (logrank p = 0.00016, HR = 0.6), GZMB (logrank p = 8.8e−06, HR = 0.54), FCGR2A (logrank p = 2.9e−06, HR = 0.53), SELL (logrank p = 0.00042, HR = 0.62), and IDO1 (logrank p = 1.1e−08, HR = 0.46), which indicated that these genes have diagnostic value (P < 0.001).

### Relationship between differentially expressed hub genes and immune cell infiltration

**Figure 6:**
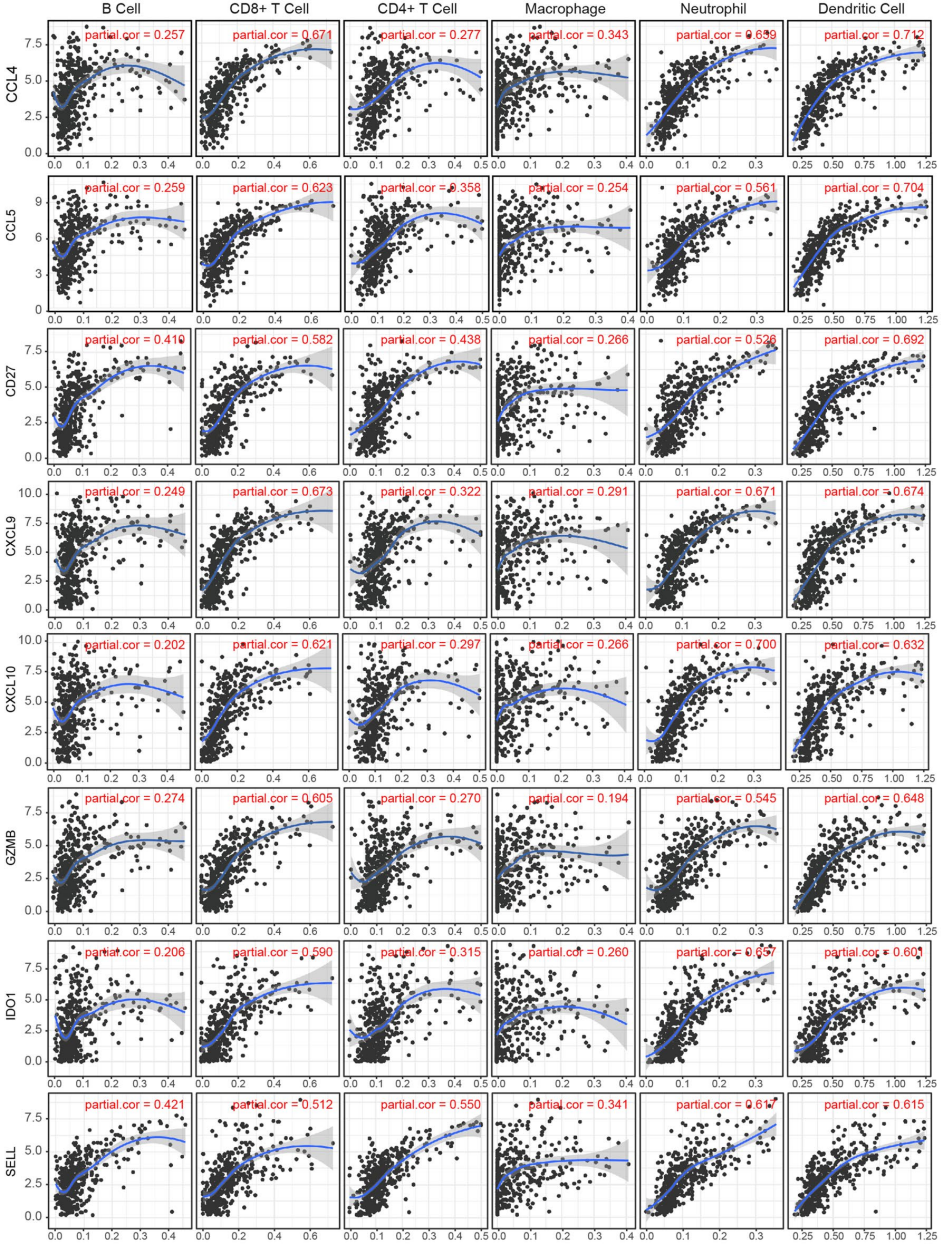
The correlation between hub genes and immune cell infiltration. The expression of hub genes was positively correlated with the infiltration level of CD8^+^ T cells, DCs and neutrophils (Cor > 0.5). In addition, SELL was also positively correlated with the infiltration level of CD4^+^ T cells. Three other immune cells (B cells, macrophages, CD4^+^ T cells) were also positively associated with these hub genes.

Functional annotation and pathway enrichment found that these differentially expressed hub genes are involved in immune responses, which suggested that they might be important in regulating immune processes during tumor progression. Therefore, we next explored the potential immune mechanisms involving the differentially expressed hub genes. The TME is a complex and dynamic ecosystem, where different cell populations coexist, mainly including tumor cells, immune cells, and supporting cells. Infiltrating immune cells play a key role in the TME. Some studies have shown that TILs are a prognostic indicator of SKCM. Therefore, we used the TIMER database to evaluate the correlation between differentially expressed hub genes and six types of invasive immune cells (neutrophils, macrophages, B cells, CD4^+^ T cells, CD8^+^ T cells, and dendritic cells) to predict their possible effects on immune cell infiltration (Fig. 6). The results showed that CTLA4, CXCL10, CCL5, CCL4, ICAM1, CXCL9, CD27, GZMB, FCGR2A, SELL, and IDO1 correlated positively with immune infiltration. Among them, FCGR2A, CTLA4, and ICAM1 had very limited correlations with the infiltration of the six immune cells (the coefficients were all less than 0.6), while CCL4, CCL5, CD27, CXCL9, CXCL10, GZMB, IDO1, and SELL are mainly related to CD8^+^ T cells, neutrophils, and dendritic cells (coefficient above 0.5). These findings suggest that these genes might be potential immune-related targets, and in-depth research on them could provide a better understanding of the TME in melanoma.

### ScRNA-Seq analysis and identification of different types of immune cells in melanoma

**Figure 7:**
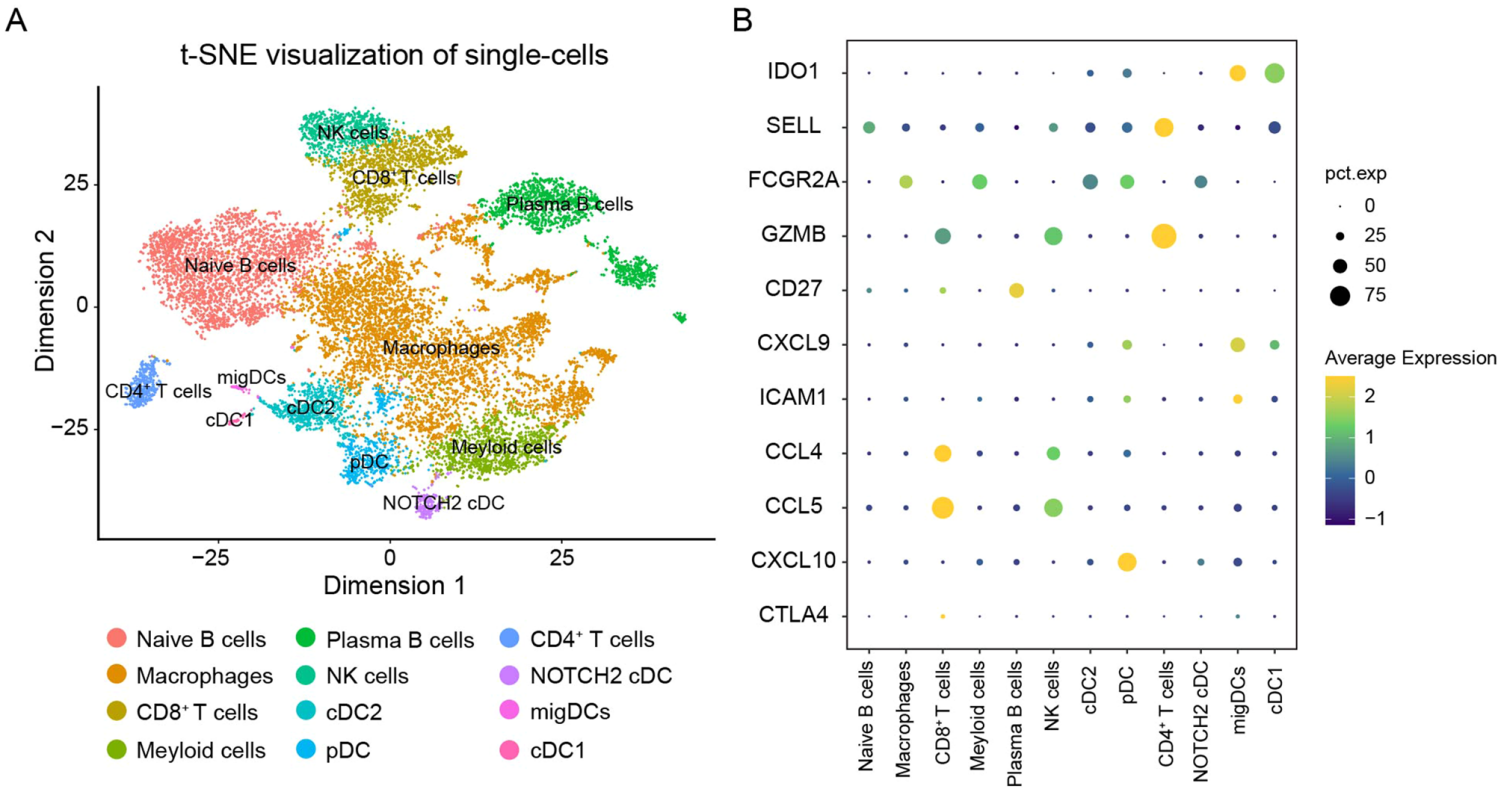
Immune cell types in SKCM were identified by single cell sequencing. (**A**) Cells were divided into 12 types of immune cells based on cell-specifically expressed markers, and the results were visualized by t-SNE dimensionality reduction clustering. (**B**) The expression of hub genes in different immune cells. *SKCM* skin cutaneous melanoma, *NK cell* natural killer cell, *cDC2* type 2 classical dendritic cell, *pDC* plasmacytoid dendritic cell, *migDC* migratory dendritic cell, *cDC1* type 1 classical dendritic cell.

Next, we used scRNA-seq data of primary melanoma (GSE123139) to analyze and identify different types of immune cells in the TME of melanoma microenvironment, using a dimensionality reduction algorithm (t-SNE) to visualize the results. t-SNE is an unsupervised machine learning algorithm that classifies cell populations into different clusters based on marker genes (Fig.S2, Supplementary file 4). We identified 12 types of cells, including macrophages, CD8^+^ T cells, B cells, and plasma cells (Fig. 7A). Further analysis of scRNA-seq data showed (Fig. 7B) that CD8^+^ T cells and NK cells highly express GZMB, CCL4, and CCL5, and about 75% of CD8^+^ T cells highly expressed CCL5. About 80% of CD4^+^ T cells highly expressed GZMB. More than 50% of plasmacytoid dendritic cells (pDCs) highly expressed CXCL10. IDO1, on the other hand, was highly expressed in migratory DC and type 1 classical dendritic cells (cDC1s).

### Integrated analysis reveals the basis of the interaction between immune cells in the TME

**Figure 8:**
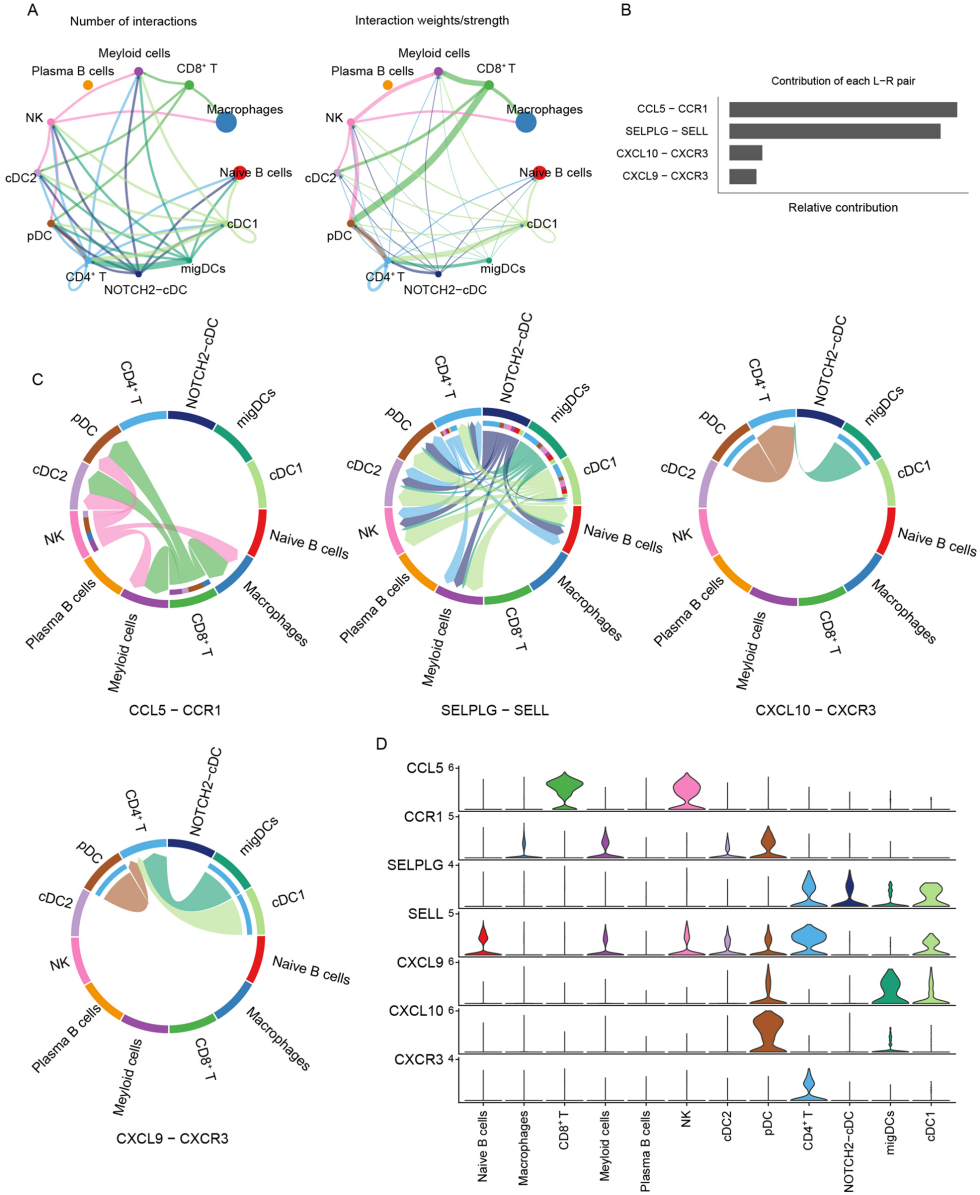
Cell communication network diagram. (**A**) The number of ligand receptor interactions detected between different cell types. (**B**) Ligand receptor contribution to the overall signaling pathway. The CCL5/CCR1 ligand receptor pair contributed the most, followed by the SELPLG/SELL ligand receptor pair. (**C**) Receptor ligand pair interactions between immune cells. (**D**) The distribution and expression level of signal genes involved in the three signal pathway networks.

To determine the potential interactions between different immune cells, we performed CellChat analysis on a data set from the GEO database (GSE123139). CellChat contains a database of receptor-ligand interactions containing 2,021 verified molecular interactions. CellChat can identify the key features of cell-to-cell communication in a given scRNA-seq data set and predict potential signaling pathways that are currently less studied. The results showed that CD8^+^ T cells, NK cells, pDCs, migratory dendritic cells (migDCs), CD4^+^ T cells and other cell populations interact closely (Fig. 8A). Next, we used CellChat to identify the interaction of immune cells in the TME. To obtain more critical cell–cell interactions in the melanoma microenvironment, we analyzed the receptor-ligand pairs related to the hub genes to explore the potential interaction between immune cells. These receptor-ligand pairs were further divided into three signaling pathways, including CCL, SELPLG, and CXCL pathways. Then, we calculated and visualized the contribution of each ligand-receptor pair to the overall signaling pathway. Among them, the ligand-receptor pairs that play a major role in the interaction between immune cells in the TME are CCL5-CCR1, SELPLG-SELL, CXCL10-CXCR3, and CXCL9-CXCR3 (Fig. 8B).

Next, we analyzed the interactions between these ligand-receptor pairs from the TME among 12 kinds of immune cells, and found that CCL5-CCR1 mainly sends signals of CD8^+^ T cells and NK Cells, which may interact with pDCs, macrophages, cDC2s, and myeloid cells via the interaction of CCL5 and CCR1. The SELPLG-SELL ligand-receptor pair is the main interaction among CD4^+^ T cells, NK cells, myeloid cells, and naïve B cells. Furthermore, NOTCH2-cDCs interact with pDCs, CD4^+^ T cells, cCD2s, NK cells, myeloid cells, naïve B cells, and cDC1s. Both migDCs and cDC1s interact with CD4^+^ T cells, pDCs, cCD2s, NK Cells, myeloid cells, and naïve B cells. The CXCL10-CXCR3 ligand-receptor pair is the main interaction among pDCs, migDCs, and CD4^+^ T cells, and the CXCL9-CXCR3 ligand-receptor pair is the main interaction among pDCs, cDC1s, migDCs, and CD4^+^ T cells (Fig. 8C). We also used a violin plot to visualize the signal gene expression distribution in the three signaling pathways inferred by CellChat. The results showed that CCL5 is mainly expressed on CD8^+^ T and NK cells, and its corresponding receptors are mainly in macrophages, myeloid cells, cCD2s, and pDCs. SELPLG is mainly expressed on CD4^+^ T cells, NOTCH2-cDCs, migDCs, and cDC1s, and its corresponding receptor, SELL, is mainly expressed on naïve B cells, myeloid cells, NK cells, cCD2s, pDCs, CD4^+^ T cells, and cCD1s. CXCL9 is mainly expressed on pDCs, migDCs, and cDC1s, CXCL10 is mainly expressed on pDCs and migDCs, and their corresponding receptor, CXCR3, is mainly expressed on CD4^+^ T cells (Fig. 8D).

### Relationship between the critical genes and immune cell infiltration in the melanoma tumor microenvironment

**Figure 9:**
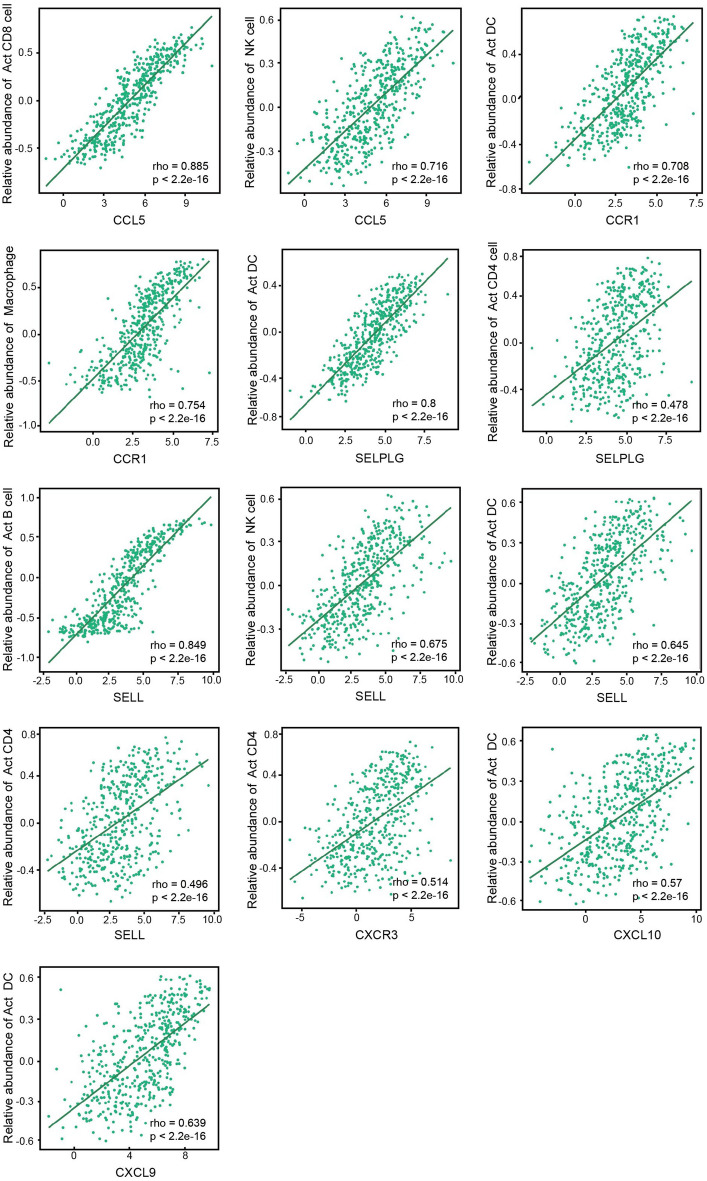
Associations of the CCL5, CCR1, SELPLG, SELL, CXCR3, CXCL10, CXCL9 expression level with immune cells in SKCM from TISIDB database.

Finally, we validated the correlation between immune cells in the melanoma tumor microenvironment with 4 ligand-receptor pairs through TISIDB, an online repository of large human cancer datasets (Fig. 9). The results showed that, basically consistent with the previous results, the CCL5 expression was positively correlated with the cellular abundance of CD8^+^ T cells (r = 0.885) and NK cells (r = 0.716), and the expression of CCR1 was associated with activated DCs (r = 0.708) and macrophages (r = 0.754), indicating that the CCL5/CCR1 ligand-receptor pair may play a key role in the interaction of CD8^+^ T cells with DCs and macrophages in the tumor microenvironment as well as the interaction of NK cells with DCs and macrophages. At the same time, the expression of SELPLG was significantly correlated with the abundance of activated DCs (r = 0.8), and relatively correlated with the abundance of CD4^+^ T cells (r = 0.478), indicating that SELPLG may have potential connection with the function of activated DC and CD4^+^T cells. The receptor corresponding to SELPLG, SELL, was also associated with activated B cells (r = 0.849), NK cells (r = 0.675), activated DCs (r = 0.645) and CD4^+^ T cell (r = 0.496) abundance which suggested that SELL on activated B cells, NK cells may interact with SELPLG on the surface of activated DCs. The chemokine receptor CXCR3 was associated with the abundance of CD4^+^ T cells (0.514), and its ligands CXCL9 (r = 0.639) and CXCL10 (r = 0.570) were both associated with activated DCs abundance. Therefore, we speculated that the ligands CXCL9 and CXCL10 on the surface of activated DCs might interact with CXCR3 on the surface of CD4^+^ T cells. These data suggested that the CCL5-CCR1, SELPLG-SELL, CXCL10-CXCR3, and CXCL9-CXCR3 ligand-receptor pairs were likely to be the key interactions between immune cells in the melanoma tumor microenvironment.

## Discussion

SKCM is one of the most life-threatening human malignancies because of its insensitivity to radiotherapy and chemotherapy, the difficulty of observing its abnormal condition at an early stage, and problems treating it at a later stage. Melanoma accounts for approximately 75% of skin cancer-related deaths^8^. The morbidity and mortality of melanoma varies greatly depending on the early detection and primary treatment in different regions^9^. Immunotherapy is a popular treatment for melanoma^10^; however, about 50% of patients do not respond to current immune checkpoint inhibitors. Therefore, finding potential predictive and efficient markers is important for tumor immunotherapy and diagnosis^11,12^.

In this study, we combined Bulk RNA-seq and scRNA-seq. Bulk RNA-seq measures the average transcript level of a cell population, which could quickly identify biological markers of a disease. In combination with scRNA-seq, cell heterogeneity could be identified, which enables in-depth study of biological structure and function. scRNA-seq technology can generate expression profiles of individual cells for analysis of heterogeneous cell populations and identification of cell types. The understanding of the phenotype of immune cells in the tumor microenvironment is essential to understand the mechanisms of cancer progression and immunotherapy.

First, we identified DEGs between primary melanoma samples and normal skin samples by analyzing datasets GSE15605 and GSE114445 from the GEO database, and the overlapping DEGs in both datasets were analyzed for functional annotation and pathway enrichment. The GO and KEGG results showed that the DEGs were closely related to tumor immunity, suggesting them as novel immune targets for melanoma treatment. We further identified and validated the hub genes among the DEGs. We finally screened genes significantly associated with overall survival, including IDO1, SELL, FCGR2A, GZMB, CD27, CXCL9, ICAM1, CCL4, CCL5, CXCL10, and CTLA4, for subsequent analysis.

Immune infiltrating cells in the TME have been shown to be very important in the antitumor immune response, including cytotoxic T cells, helper T cells, B/plasma cells, and macrophages/monocytes^13^. Identifying expression of hub genes in the specific immune cell types of the TME will help to better understand the underlying mechanisms by which immune cells promote and counteract tumor progression. In our study, we also found a strong positive correlation between the above-mentioned hub genes and the level of immune infiltration of six types of invasive immune cells (including B cells, CD4^+^ T cells, CD8^+^ T cells, neutrophils, macrophages, and dendritic cells). This implied a strong association of these hub genes with immune cells in the SKCM TME, which was subsequently validated using scRNA-seq datasets.

CellChat can quantitatively infer and analyze intercellular communication networks from scRNA-seq data^7^. Most of its ligand receptor interactions are based on the KEGG signal pathway database. After the user inputs the cell gene expression matrix, CellChat models the probability of intercellular communication and allows the user-defined ligand receptor pair to update the CellChatDB. We updated the CellChatDB by selecting the receptor ligand pairs corresponding to the hub genes. Our results showed the number and intensity of interactions between immune cells in the TME. The ligand receptor pairs that play an key role in the interaction between immune cells in the TME are CCL5-CCR1, SELPLG-SELL, CXCL10-CXCR3, and CXCL9-CXCR3.

The CCL5/CCR1 axis has been found to play a regulatory role in tumorigenesis and progression in several studies. Tumor growth is controlled by the recruitment of immunocompetent host cells such as T cells, NK cells, and DCs^14^. Studies have shown that CCL5 directly affects the transport of immune cells and participates in anti-tumor immune response. CCL5 could recruit NK cells and cDC1s to infiltrate the TME^15,16^. Meanwhile, the expression level of CCL5 at the tumor site determined the effectiveness of the antitumor response, which may be related to the increased number of NK cells and CD8^+^ T cells at the tumor site. CCL5 belongs to the CC chemokine family and may activate several chemokine receptors, including CCR1, CCR3, CCR4 and CCR5, and regulates the expression and secretion of activated normal T cells^17^. However, at present, the nature of the interaction between CCL5 and immune cells in the TME has not been determined. The scRNA-seq analysis showed that the expression level of CCL5 was high on NK cells and CD8^+^ T cells (Fig. 7B); therefore, we hypothesized that CCL5 may be the key node for two-way communication between NK cells or CD8^+^ T cells and other TME immune cells. CCR1, the receptor of CCL5, can interact with a variety of chemokines, and has the highest binding affinity with CCL3 and CCL5. CCR1 or CCR3 can bind to CCL5 to mediate its activity^18,19^. As shown in Fig. 8B, the CCL5-CCR1 ligand-receptor pair has the highest contribution to immune cell interaction, and mainly mediates the interactions among macrophages, myeloid cells, cDC2s and pDCs. And the TISIDB database verified that CCL5 expression was positively correlated with the celluar abundance of CD8^+^ T cells and NK cells, and the expression of CCR1 was positively correlated with the cellular abundance of activated DCs and macrophages. Therefore, we propose that macrophages, cDC2 and pDC may interact with the CCL5 ligand on NK cells in TME through CCR1 receptors on their surfaces.

CellChat analysis revealed the contribution of the SELPLG-SELL pathway to the interaction between the TME and immune cells. Figure 8D shows that the SELPLG-SELL pathway might affect the interaction between CD4^+^ T cells and other immune cells in the TME. SELPLG is also known as the adhesion molecule P-selectin glycoprotein ligand-1 (PSGL-1). In melanoma models with T cell dysfunction, PSGL-1 deficiency leads to programmed cell death-1 (PD-1) downregulation, an improved T cell response, and tumor control, and PSGL-1 acts as a negative regulator of CD4^+^ T cells in a variety of diseases, including cancer. Scholars have suggested that blocking PSGL-1 in CD4^+^ T cells might represent a new cancer treatment strategy^20^. PSGL-1 is the ligand of all selectins (P-, L- and E-selectins); therefore, reducing the interaction between selectins and PSGL-1 might also improve T cell responses^21^. P-selectin on DCs could give rise to the tolerance phenotype, which could suppress T cells^22^. Another study has shown that cDC1s play an important role to activate the CD4^+^ and CD8^+^ T Cell response to tumors^23^; however, at present, it is unclear whether PSGL-1 on T cells and/or cDC1s contributes to T cell activation. CellChat analysis showed that CD4^+^ T cells and cDC1s might interact through SELPLG-SELL (Fig. 8D). The TISIDB results showed that SELPLG was related to the abundance of CD4^+^ T cells in tumor-infiltrating lymphocytes, while SELPLG and its receptor SELL were also closely related to the cellular abundance of activated DCs^24^.

CXC Chemokine Receptor 3 (CXCR3) is considered a type 1 T helper cell (Th1) receptor. CXCR3 can bind to its ligands CXC motif chemokine (CXCL) 9, CXCL10, and CXCL11^25,26^. Studies have shown that the chemotactic function of CXCR3 plays an important role in autoimmune diseases and cancers^27,28^. CellChat analysis showed that CXCR3 is highly expressed on CD4^+^ T cells. The CXCL10-CXCR3 and CXCL9-CXCR3 pairs might be the key interactions between CD4^+^ T cells and pDCs, migDCs, and cDC1s in the TME. The CXCL9-CXCR3 and CXCL10-CXCR3 axes mainly regulate the migration, differentiation, and activation of immune cells. Immune cells can be recruited through chemotaxis mediated by CXCL9-CXCR3 or CXCL10-CXCR3. Studies have shown that tumor-resident cDC1s are the main source of CXCL9 and CXCL10^29^, and CXCL10 is a candidate for cancer immunotherapy. There is a strong correlation between insufficient expression of CXCL10 and poor prognosis at tumor sites in various human cancers^30^. In addition, CXCL10 can induce CD8^+^ and CD4^+^ effector T cells to the tumor site and enhance their function^31^. In the TISIDB results, CXCR3 was shown to be closely related to the cellular abundance of CD4^+^ T cells, while CXCL9 and CXCL10 were closely related to the cellular abundance of activated DCs. Therefore, combining the results of CellChat and TISIDB analysis, we hypothesized that CXCL10-CXCR3 and CXCL9-CXCR3 are key points of interaction between CD4^+^ T cells and other immune cells in TME.

Tumor-infiltrating immune cells play a critical role in tumor progression and prognostic assessment. Further exploration of the interactions between T cell subsets and other infiltrating immune cells could help to better understand melanoma progression and improve its prognosis. All the above results suggested that the ligand-receptor pairs CCL5-CCR1, SELPLG-SELL, CXCL10-CXCR3, and CXCL9-CXCR3 play an important role in the communication between immune cells in the TME. Our findings form the basis for future research. These biomarkers have prognostic significance and could be effective therapeutic targets for melanoma treatment. Understanding their complex roles in tumor biology will help improve the efficacy of cancer immunotherapy strategies, induce durable host anti-tumor immunity, and provide new ideas for SKCM immunotherapy.

## Materials and methods

### Data collection and processing

The GEO database (http://www.ncbi.nlm.nih.gov/geo/) is a public database used to host high-throughput microarray and next-generation sequence functional genomic datasets^32^. We downloaded expression profiles of patients with SKCM with clinical data from the GEO database. For this part of the study, we selected the datasets GSE15605 and GSE114445^33,34^. The data of GSE15605 were obtained with the GPL570 Platforms (Affymetrix Human Genome U133 Plus 2.0 Array) by Vanderbilt University, and came from 46 primary melanoma samples, 12 regional or distant metastases, and 16 normal skin samples. In this dataset, we only selected 46 primary melanoma samples and 16 normal skin samples for subsequent analysis. Similarly, the data of GSE114445 were based on the GPL570 platform (Affymetrix Human Genome U133 Plus 2.0 Array). We analyzed 16 primary melanoma tissues and 6 normal skin tissues in the GSE114445 dataset. We also selected the dataset with accession number GSE123139, which includes data from single-cell transcriptional analysis of immune cells from human melanoma tumors^35^. The samples were obtained from public databases and the study was carried out in accordance with relevant guidelines/regulation. The statement of ethics approval and informed consent were not needed for this study.

### Identification of DEGs

GEO2R (https://www.ncbi.nlm.nih.gov/geo/geo2r/) is an online analysis tool based on the limma package^36^, which enables users to identify DEGs in one or more datasets. We used GEO2R to separately screen DEGs between PM tissue samples and NS tissue samples from the GSE15605 and the GSE114445 datasets. DEGs were defined using the threshold of |log fold-change (FC)|> 1 and an adjusted P-value (adj.P.Val) ≤ 0.05. When the expression difference of a gene between normal tissue and tumor tissue meets this criterion, the differential expression of the gene is judged to be statistically significant. And when logFC is negative, it means low expression of genes in tumor tissues compared to normal tissue groups, and conversely, it means high expression. We next used the R software to extract the common DEGs between the two datasets and visualized them using volcano plots, heat maps, and Venn plots^37^.

### Gene ontology (GO) and kyoto encyclopedia of genes and genomes (KEGG) analysis

GO (gene ontology) databases define biological processes (BP), cellular components (CC), and molecular functions (MF) based on gene products and are used widely to interpret genomes^38^. The KEGG database links genomic and functional information, allowing users to analyze gene functions^39–41^. DAVID (https://david.ncifcrf.gov/) is a free online tool that extracts biological significance from large lists of genes or proteins, providing users with functional annotation and visualization^42,43^. In this study, we used DAVID f to obtain GO functional enrichment analyses and enriched KEGG pathways of the DEGs. P < 0.05 was considered statistically significant and the results were visualized using ggplot2 in the R package.

### Acquisition of hub genes by PPI network analysis

The STRING database (https://string-db.org/, version: 11.5) is used to predict PPI networks from DEGs and to analyze the interaction between proteins^44^. We used the STRING website to construct the PPI network from DEGs, using a minimum interaction score of 0.4. The plug-in cytoHubba in Cytoscape (version 3.7.2) was used to visualize the protein–protein interaction (PPI) network, and the identified the central genes by maxinmal clique centrality (MCC, one of the 12 methods to explore important nodes in biological networks) computing method^45,46^.

### Expression verification and survival analysis of the hub genes

Gene Expression Profiling Interactive Analysis (GEPIA) is a web-based comprehensive analysis tool, including RNA sequencing data of tumor and normal samples from The Cancer Genome Atlas (TCGA) and genotype tissue expression projects (GTEx). GEPIA can analyze changes in gene expression in tumor and normal tissues, and provide gene interactions, functions, and prognostic data^47^. We used the GEPIA data to determine the differential expression and prognostic predictive significance of hub genes in normal and cancer tissues. A p value < 0.05 was considered statistically significant.

### Tumor immune infiltration analysis

The Tumor Immune Estimation Resource database (TIMER) (https://cistrome.shinyapps.io/timer) is an easy-to-use web interface to study the molecular characteristics of tumor immune interaction^48^. TIMER can estimate the abundance of six types of infiltrating immune cells from gene expression profiles. To investigate the immune infiltration of genes in SKCM, we used the TIMER database to assess whether the level of tumor infiltrating lymphocytes (TILs) correlated with gene expression in SKCM. A p value < 0.05 was considered to be statistically significant.

### Single cell sequencing data processing

This study used the original gene expression matrix from the dataset GSE123139, which includes single-cell RNA sequencing (scRNA-seq) data from 25 patients with melanomas, with the aim of applying scRNA-seq data to dissect the immune cell composition and molecular features of the tumor microenvironment in SKCM. The quality control (QC) process used the R package Seurat (version 4.0.4)^49^. The data filtering indicators were as follows: 1. The number of genes is greater than 300. 2. Cells with RNA greater than 1000 and less than 20,000. 3. Less than 12.5% mitochondrial genes. 4. Ribosomal genes accounted for more than 0.3%. 5. Less than 3% erythrocyte genes. At the end of QC, the cell populations were annotated according to marker genes, and markers were identified as major immune cell types and visualized with the dimensionality reduction algorithm t-distributed stochastic neighbor embedding (t-SNE)^50^.

### Inference and analysis of intercellular communication using CellChat

scRNA-seq data contains gene expression information that could be used to infer intercellular communication. CellChat is a tool that can quantitatively infer and analyze intercellular communication networks from scRNA-seq data^7^. We applied CellChat analysis to the scRNA-seq data of the SKCM samples. CellChat contains a database of receptor-ligand interactions. To obtain more critical cell–cell interactions in the melanoma microenvironment, we selected receptor-ligand pairs related to the hub genes for further analysis, aiming to explore the potential interactions between immune cells.

### Database applied to analyze genes and immune cell infiltration correlation

The TISIDB (http://cis.hku.hk/TISIDB/index.php) is an online storage database that collects a large number of human cancer data sets, and calculates a variety of genes and immune characteristics (such as lymphocytes, immune modulators and chemokines, etc.). The correlation between the four receptor-ligand pairs (CCL5-CCR1, SELPLG-SELL, CXCL10-CXCR3, and CXCL9-CXCR3) and immune cell infiltration in the melanoma tumor microenvironment was verified through the TISIDB.

## Conclusions

In this study, we used bioinformatics to identify and verify the key genes related to the prognosis of SKCM, and explored the prognostic value of these genes. In addition, functional enrichment analysis showed that these genes are involved in the tumor immune response. We further predicted that the ligand-receptor pairs CCL5-CCR1, SELPLG-SELL, CXCL10-CXCR3, and CXCL9-CXCR3 might be the key points of communication between immune cells, revealing the potential cellular communication basis of CD8^+^ T cells in the tumor microenvironment and contributing to a better understanding of melanoma development.

## Supplementary Information

Supplementary Information 1.

Supplementary Information 2.

Supplementary Information 3.

Supplementary Information 4.

Supplementary Information 5.

## Data Availability

The datasets generated and analyzed during the current study are available at GEO database (https://www.ncbi.nlm.nih.gov/geo/), including GSE15605 (https://www.ncbi.nlm.nih.gov/geo/query/acc.cgi?acc=GSE15605), GSE114445 (https://www.ncbi.nlm.nih.gov/geo/query/acc.cgi?acc=GSE114445) and GSE123139 (https://www.ncbi.nlm.nih.gov/geo/query/acc.cgi?acc=GSE123139).
