# Supplementary material for: Integrated analysis reveals the pivotal interactions between immune cells in the melanoma tumor microenvironment
Source: Sci Rep. 2022 Jun 16;12:10040. doi: 10.1038/s41598-022-14319-2 (PMC9203818; doi:10.1038/s41598-022-14319-2)
Supplement: Supplementary file 5 — Supplementary Information 5. [file 41598_2022_14319_MOESM5_ESM.docx]

Integrated analysis reveals the pivotal interactions between immune cells in the melanoma tumor microenvironment

Jiawei Chen^1, †^, Shan Hu^1, 2, †^, Huiqi Wang^1, †^, Tingxiu Zhao^2^, Yue Song^1, 3^, Xueying Zhong^1^, Qingling Luo^1^, Mansi Xu^1^, Lina He^1^, Qiugu Chen^1, 3^, Biaoyan Du^2, *^, Jianyong Xiao^1, 3, *^, Kun Wang^1, 2, *^

^1^ Research Center of Integrative Medicine, School of Basic Medical Sciences, Guangzhou University of Chinese Medicine, Guangzhou 510006, China.

^2^ Department of Pathology, Guangzhou University of Chinese Medicine, Guangzhou 510006, China.

^3^ Department of Biochemistry, Guangzhou University of Chinese Medicine, Guangzhou 510006, China.

*** Correspondence:**wangkun@gzucm.edu.cn (K.W.), jianyongxiao@gzucm.edu.cn (J.X.) or dubiaoyan@gzucm.edu.cn (B.D.).

† These authors contributed equally to this work.

Supplementary Figures

**Figure S1.** Differential genes with MCC score ≥ 10000 were screened to construct a PPI network.


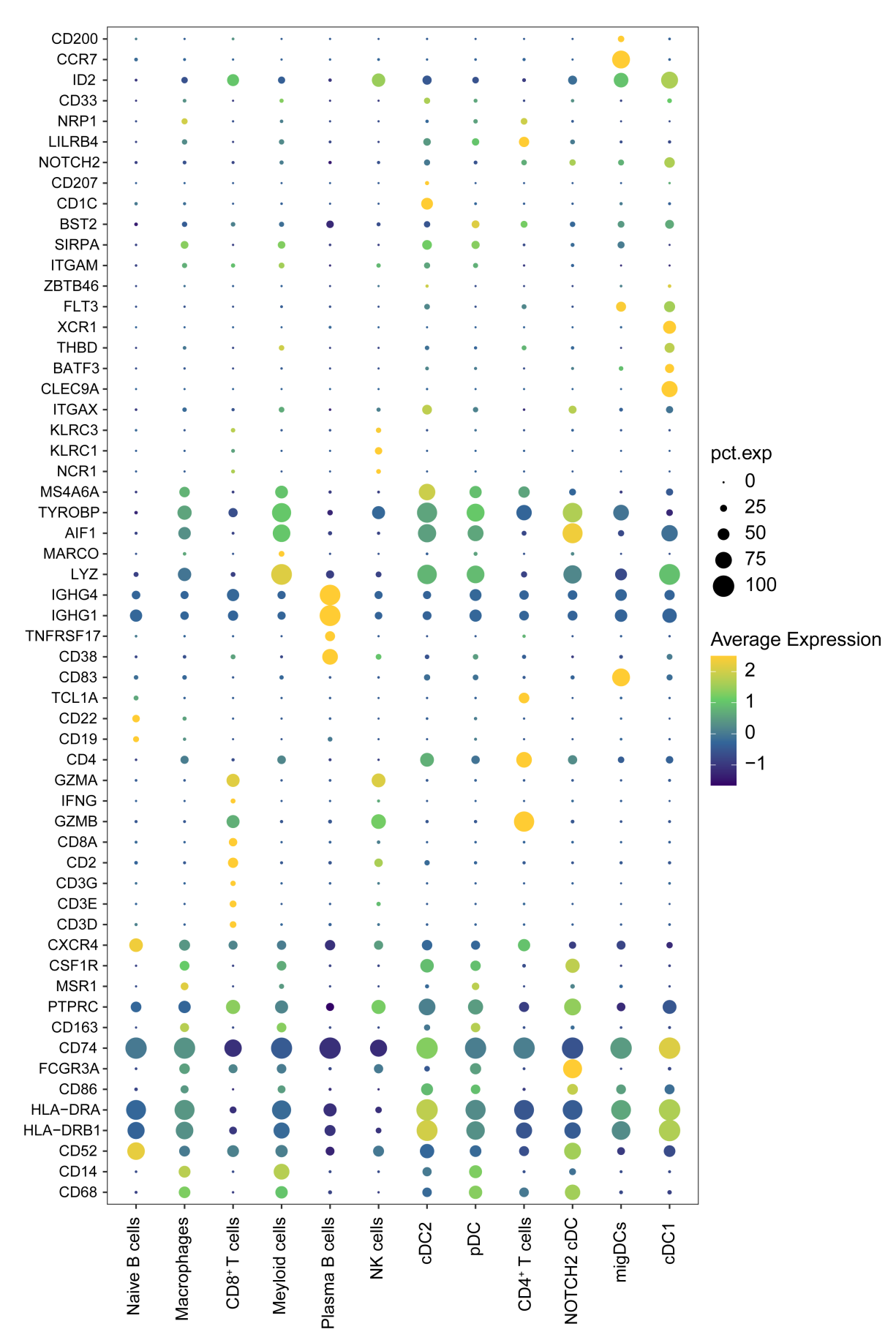


**Figure S2.** The expression levels of marker genes used for cell annotation in different immune cells.
